# Evaluating climate-related financial policies’ impact on decarbonization with machine learning methods

**DOI:** 10.1038/s41598-025-85127-7

**Published:** 2025-01-11

**Authors:** Paola D’Orazio, Anh-Duy Pham

**Affiliations:** 1 Chair of Economics, Faculty of Economics and Business Administration, Chemnitz University of Technology, Thüringer Weg 7, 09126 Chemnitz, Germany; 2 Joint Lab Artificial Intelligence and Data Science, Osnabrück University, 49074 Osnabrück, Germany

**Keywords:** Climate-related financial policies (CRFPs), Decarbonization, Renewable energy production (REP), Policy sequencing score (PSS), Bindingness-weighted policies, Institutional and Economic contexts, Environmental social sciences, Climate-change mitigation, Climate-change policy, Environmental economics

## Abstract

This study examines how Climate-Related Financial Policies (CRFPs) support decarbonization and renewable energy transitions across 87 countries from 2000 to 2023. Using the Policy Sequencing Score (PSS) and a bindingness-weighted adoption indicator, it explores the relationships between CRFPs, CO2 emissions, and Renewable Energy Production (REP) across diverse economic and institutional contexts. Findings reveal significant variation in outcomes. Advanced economies and OECD countries leverage structured policies and robust institutions to achieve steady emissions reductions and REP growth, with diminishing returns at higher policy intensities. Emerging Markets and Developing Economies (EMDEs) face institutional and structural constraints but show strong responsiveness to targeted policies, particularly in Sub-Saharan Africa and South Asia, where renewable energy growth potential is notable. Regions such as Latin America and East Asia display mixed trends, reflecting unique challenges and opportunities. Binding policies prove essential for environmental outcomes, particularly in institutionalized settings, while EMDEs require capacity building and international cooperation to address barriers. This study highlights the importance of tailoring CRFPs to specific contexts, emphasizing policy sequencing, enforcement, and capacity building. By identifying global and regional variations, the findings provide actionable insights for aligning financial systems with climate goals, fostering a sustainable low-carbon transition, and addressing equity challenges.

## Introduction

Climate-related financial policies (CRFPs) are essential in aligning the financial sector with environmental objectives and promoting investments in green initiatives^1,2^. These policies serve two critical purposes. First, they enhance the financial sector’s capacity to manage climate-related risks, ensuring stability in the face of environmental challenges^3–6^. Second, they redirect capital flows toward sustainable investments, reducing the carbon intensity of financial activities and accelerating the transition to a low-carbon economy^7,8^. This second role aligns with Article 2.1(c) of the Paris Agreement, which emphasizes the necessity of aligning financial flows with pathways that support low greenhouse gas (GHG) emissions and climate-resilient development^9^.

Financial authorities, such as central banks, financial supervisors, and regulators, employ several instruments to advance these goals^10–13^. Tools like stress testing, mandatory disclosures, and climate risk assessments enable policymakers to identify vulnerabilities, mitigate risks, and drive sustainable capital allocation^3,14^. This mitigates systemic risks by ensuring financial institutions remain resilient to climate disruptions^15–17^. Meanwhile, measures like green bonds and climate-related financial guidelines foster the growth of green finance by creating markets for sustainable investments^18–21^. Green bonds have proven to be an influential instrument for financing renewable energy and energy efficiency projects^22^, particularly in regions like Southeast Asia. For example, two-thirds of green bonds issued in ASEAN countries, such as Indonesia, Malaysia, and Singapore, have been directed toward renewable energy and energy efficiency initiatives. However, some proceeds are allocated to refinancing or financing projects abroad, which limits their direct contribution to local sustainability goals^23,24^. Beyond green bonds, mandatory climate risk disclosures have reshaped investment patterns, driving capital away from high-emission sectors. Evidence shows that firms with higher carbon emissions incur a “carbon risk premium” as investors increasingly demand compensation for exposure to carbon-intensive industries, reflecting the growing influence of carbon risk on capital allocation^25^.

Since the Paris Agreement, climate-related financial sector policies have gained significant traction, with 81 countries (37%), up from 43 countries in 2015. These policies, implemented by both advanced economies and emerging and developing economies—including all G20 jurisdictions - address climate change mitigation and resilience. While primarily aimed at managing climate-related transition and physical risks to ensure financial system stability, they also influence the alignment of finance with climate goals, even when this is not their direct objective^5,9^. A diverse set of policymakers has driven these policies: governments account for 30%, supervisory and regulatory authorities for 26%, central banks for 28%, and stock and securities exchanges for 9%, with some policies reflecting joint efforts^9,26^. The mandates and capacities of these authorities to integrate climate considerations vary by jurisdiction, influencing the types of policies they prioritize^27^.

Policy sequencing the order and timing of policy implementation - is critical for ensuring effective and sustainable governance^28,29^. In economic policymaking, sequencing financial reforms establishes a strong foundation for subsequent measures, reducing risks and enhancing their overall impact^30–32^. Similarly, in environmental governance, the early adoption of enabling policies—such as subsidies for green technologies—facilitates the implementation of more stringent measures, like carbon pricing, by lowering transition costs and building public and political support^33^. Research has also examined the design and impacts of climate policies, emphasizing the role of sequencing in achieving their objectives^34,35^. Moreover, crisis management demonstrates the importance of well-coordinated and timely interventions in mitigating socio-economic disruptions^36^.

Against this backdrop, this study contributes to the global discourse by addressing the impact of sequencing and enforceability of Climate-Related Financial Policies (CRFPs) on the low-carbon transition and decarbonization, which remain underexplored areas^9,13,37^. Specifically, it investigates how the sequencing, duration, and binding nature of CRFPs influence key decarbonization indicators, such as CO2 emissions and renewable energy production (REP). Using data from 87 countries over 23 years (2000–2023), this analysis examines the effects of financial policies across diverse economic, institutional, and regional contexts, enabling the identification of broad trends and the contextualization of policy impacts within specific settings. The inclusion of both developed and developing economies allows for a comprehensive analysis of CRFPs across varying institutional, economic, and regulatory landscapes. Developed economies provide a benchmark for assessing mature policy frameworks while developing economies highlight the unique challenges and potential for innovative policy approaches in less institutionalized contexts. This dual focus enables the identification of globally relevant patterns and region-specific nuances in policy impacts.

To advance this understanding, the study introduces the Policy Sequencing Score (PSS) and cumulative bindingness-weighted adoption as policy design and implementation measures, providing insights into their distinct roles in shaping environmental outcomes. This study employs machine learning (ML) methods^38–40^ to investigate the complex relationships between CRFPs and environmental outcomes, such as CO2 emissions and renewable energy production. ML provides key advantages by capturing nonlinear relationships and interactions, enabling a nuanced analysis of policy impacts across diverse economic and institutional contexts. Tools such as SHAP (Shapley Additive Explanations) enhance interpretability by identifying the relative importance of features like policy sequencing and bindingness in shaping environmental outcomes. While the study does not aim to establish causality, it offers robust predictive insights and uncovers patterns consistent with theoretical expectations. These findings help policymakers anticipate the potential effects of CRFPs and design interventions tailored to specific contexts. By revealing heterogeneities across regions and country groups, the analysis provides evidence to support the alignment of financial systems with global climate goals.

## Climate-related financial policies: evolution, implementation patterns, and decarbonization pathways

### Mapping climate-related financial policy evolution: sequences, transitions, and policy sequencing scores

In this study, a policy sequence refers to the chronological progression of climate-related financial policies (CRFPs), including Green Bonds (GB), Green Credit Allocation Policies (GCA), Green Prudential Policies (GPP), Green Financial Guidelines (GFG), and Other Disclosure Requirements (OGD). By analyzing these sequences, we identify patterns and transitions that reveal their implications for decarbonization and financial stability.

**Fig. 1:**
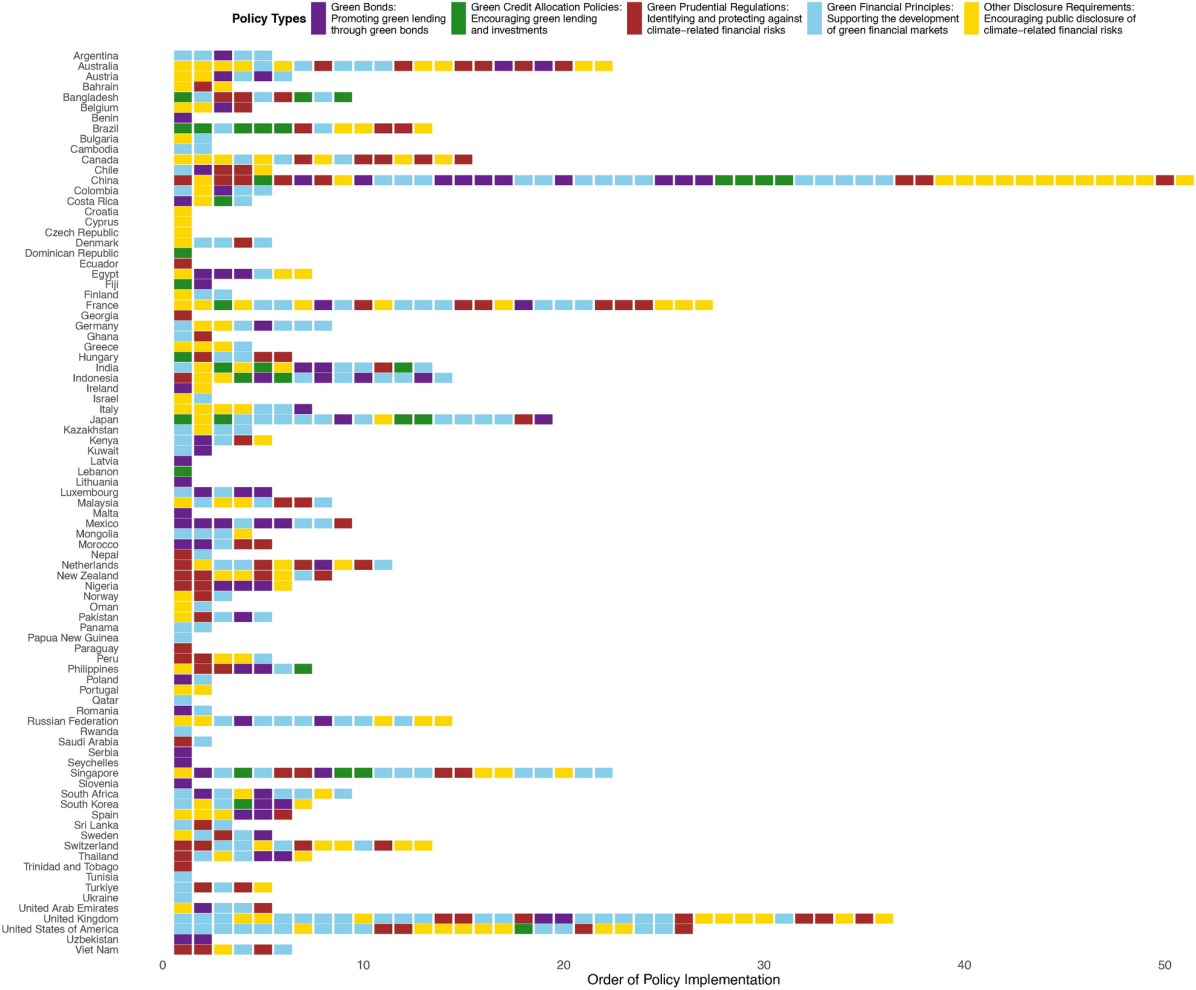
Policy implementation sequences across countries. The order of policy implementation for various countries is categorized into five policy types: *Green Bonds (GB)*, promoting green lending through bonds; *Green Credit Allocation Policies (GCA)*, encouraging green lending and investments; *Green Prudential Regulations (GPP)*, identifying and protecting against climate-related financial risks; *Green Financial Principles (GFG)*, supporting the development of green financial markets; and *Other Disclosure Requirements (OGD)*, encouraging public disclosure of climate-related financial risks.

As shown in Fig. 1, countries with longer sequences demonstrate a structured and diverse approach to policy implementation. For instance, Brazil, China, Indonesia, and Vietnam prioritize policies like GCA and GPP early in their sequences, establishing a stable financial system before adopting market-based instruments such as green bonds and guidelines. This strategic progression highlights their focus on balancing stability with green investment. Advanced economies like the United Kingdom and France exhibit extensive sequences, starting with OGD to enhance transparency and investor confidence, followed by green guidelines and market-oriented measures like green bonds. In contrast, countries with shorter sequences usually focus on one or two policy types, reflecting targeted strategies often constrained by limited resources or specific priorities.

**Fig. 2:**
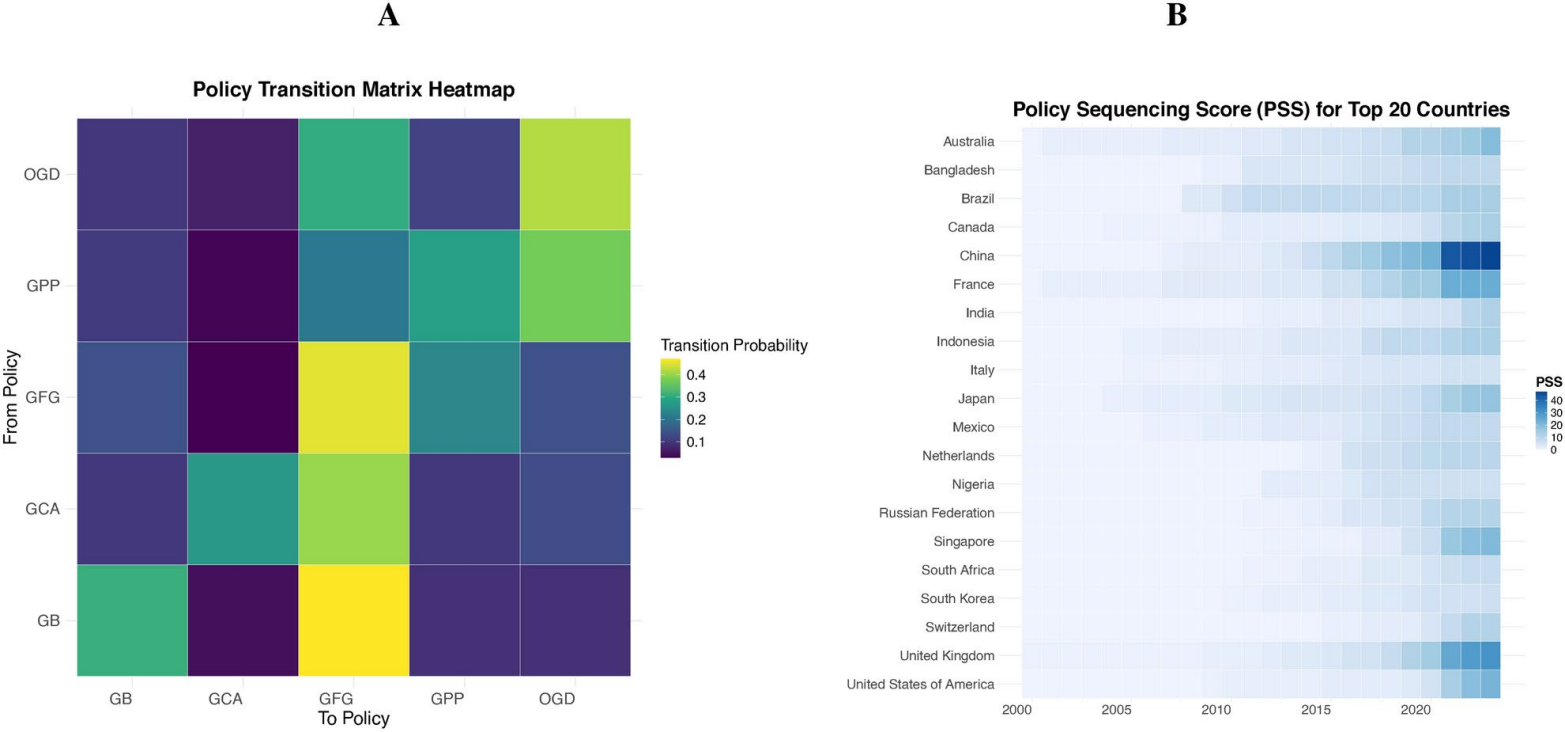
(**A**) Policy Transition Matrix (left panel): probabilities of transitioning from one policy type (*From Policy*) to another (*To Policy*). The highest transition probabilities are indicated by the brightest colors (green and yellow) in the heatmap, representing the highest transition probability among all combinations of policy types. (**B**) Policy Sequencing Scores for Top 20 Countries (right panel): evolution of Policy Sequencing Scores (PSS) over time for the top 20 countries (y-axis) based on their average scores. Darker shades of blue represent higher PSS values, revealing differences in policy intensity and trends across countries and years.

To further understand the evolution of CRFPs, we analyze the likelihood of transitioning from one policy type to another, as depicted in the Transition Probability Matrix (TPM) in **Panel A** of Fig. 2. The matrix highlights the probabilities of moving from one policy type (rows) to another (columns). The transitions with the highest probabilities include from GPP (Green Prudential Policies) to GFG (Green Financial Guidelines), as indicated by the brightest cell, followed by notable transitions such as from GCA (Green Credit Allocation) to GFG and from OGD (Other Green Disclosure) to GFG. These patterns suggest that GFG often serves as a common follow-up policy type, reflecting its likely role in complementing or reinforcing other policy measures within the CRFP framework.

**Panel B** in Fig. 2 illustrates the evolution of Policy Sequencing Scores (PSS) for the top 20 countries from 2000 to 2023. The PSS quantifies the systematic adoption and structuring of CRFPs, revealing significant disparities across countries. Advanced economies like Australia, Japan, France, and the United Kingdom maintain consistently high PSS values, reflecting early leadership and sustained efforts to integrate green finance into policy frameworks. EMDEs like Brazil, China, and Indonesia also show early and notable growth of policy sequences. China, in particular, has experienced rapid acceleration since 2015, signaling a concentrated push toward comprehensive policy frameworks. In contrast, countries like Canada, Italy, and Singapore display late and slower progress.

### How policy sequencing and bindingness-weighted adoption drive climate transitions

To study how the sequencing, duration, and binding nature of CRFPs influence key decarbonization indicators, such as CO2 emissions and renewable energy production (REP), we rely on the Policy Sequencing Score (PSS) and Cumulative Policy Index $\times$ Bindingness (CumBind). They are complementary metrics that capture different aspects of climate-related financial policy (CRFP) adoption. The PSS evaluates how closely a country’s policy adoption aligns with typical global sequences. It is calculated by summing the conditional frequencies of policies adopted up to a given year, where conditional frequencies represent the likelihood of one policy preceding another globally. A higher PSS indicates that a country’s policies are implemented in a structured and globally consistent order, reflecting strategic alignment with best practices. The CumBind accounts for both the quantity and enforceability of policies. It is computed by summing the bindingness-weighted policies adopted over time, where Bindingness reflects a policy’s enforceability (e.g., mandatory, voluntary, or non-binding). This index highlights the intensity and effectiveness of a country’s policy framework by incorporating the strength of each policy.

Through SHAP summary plots and fitted curves, in this section, we assess the contributions of these policy metrics across four country groups: Emerging Markets and Developing Economies (EMDEs), Advanced Economies (ADV), G20, and OECD countries. The results reveal how variations in economic structures, institutional capacity, and policy frameworks influence the impact of policy measures in achieving decarbonization.

**How policy sequencing and cumulative bindingness shape** ${{\textbf {CO}}}_2$ **emissions: evidence across institutional contexts** We propose that countries with longer sequences (PSS) of climate-related financial policies (CRFPs) and higher implementation stringency—reflected in elevated CumBind scores—are associated with significant increases in renewable energy production (REP) and reductions in CO2 emissions. This proposition is supported by evidence from other policy domains^41,42^, which demonstrates that extended policy sequences foster stable business environments and stimulate greater investment in clean energy technologies, both of which are critical for transitioning to renewable energy systems and mitigating emissions.

To explore whether countries with longer policy sequences (high PSS) and higher implementation stringency (high CumBind) align with these environmental outcomes, we utilize machine learning models to uncover patterns and relationships between CRFPs and renewable energy production or CO2 emissions reductions. By leveraging SHAP values, we gain interpretable insights into the relative contributions of policy characteristics to these outcomes.

**Fig. 3:**
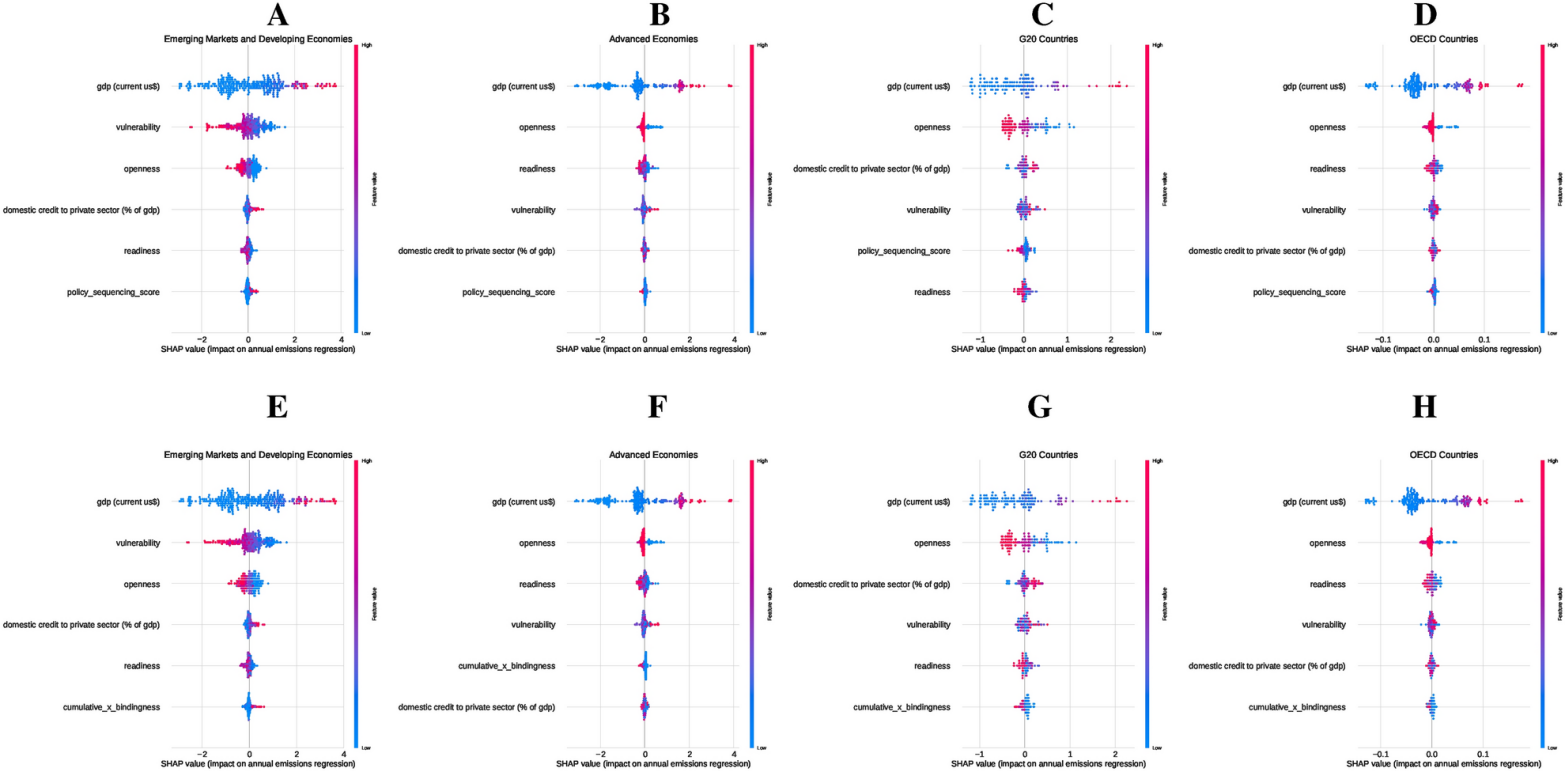
Impact of Policy Sequencing Score (Panels A–D) and Cumulative Adoption with Bindingness (Panels E–H) on ${{\textbf {CO}}}_2$ Emissions across country groups, measured using SHAP summary plots. Panel A (EMDEs) highlights GDP, vulnerability, and openness as dominant factors, with PSS contributing modestly. Panel B (Advanced Economies) shows GDP, openness, and readiness as the most influential factors, with PSS playing a significant role in reducing emissions. Panel C (G20 countries) emphasizes GDP, openness, and domestic credit, with PSS aiding consistency in diverse economies. Panel D (OECD countries) identifies GDP, openness, and readiness as key drivers, with PSS providing steady contributions. In Panels E-H, cumulative bindingness gains prominence: in Panel E (EMDEs), GDP, vulnerability, and openness dominate, but cumulative bindingness adds meaningful impact; Panel F (Advanced Economies) highlights its strong role alongside GDP and readiness; Panel G (G20 countries) shows its influence tapering after initial contributions; and Panel H (OECD countries) shows cumulative bindingness providing modest but consistent support in advanced institutional contexts.

Figure 3 presents SHAP summary plots illustrating the impact of PSS and CumBind on $\hbox {CO}_2$ emissions across the four country groups. The SHAP values measure the direction and magnitude of each feature’s contribution to $\hbox {CO}_2$ emissions, ranking them by importance.

In EMDEs (Panel A), GDP, vulnerability, and openness are the primary drivers of CO2 emissions, with PSS playing a secondary role by stabilizing policy environments. Vulnerability highlights the significant influence of external risks on emissions outcomes. When considering CumBind (Panel E), GDP, vulnerability, and openness remain dominant factors, while CumBind has a modest but meaningful impact, underscoring the importance of policy enforcement despite institutional constraints. In Advanced Economies (Panel B), GDP, openness, and readiness emerge as the main determinants of emissions, with PSS significantly enhancing emissions reductions by fostering stable conditions for long-term investments. Similarly, in the context of CumBind (Panel F), GDP, openness, and readiness lead the way, with CumBind playing a critical role in transforming policies into effective emissions reductions. In G20 countries (Panel C), GDP, openness, and domestic credit are key contributors to CO2 emissions, while PSS ensures consistent policy frameworks across these diverse economies. For CumBind (Panel G), GDP, openness, and domestic credit remain dominant, with CumBind providing secondary but relevant support, especially in countries with varying institutional capacities. For OECD countries (Panel D), GDP, openness, and readiness are the leading factors, with PSS offering stable support for emissions reductions in institutionalized settings. When examining CumBind (Panel H), GDP, openness, and readiness continue to play a central role, while CumBind facilitates emissions reductions by strengthening policy enforcement within mature institutional frameworks.

**Fig. 4:**
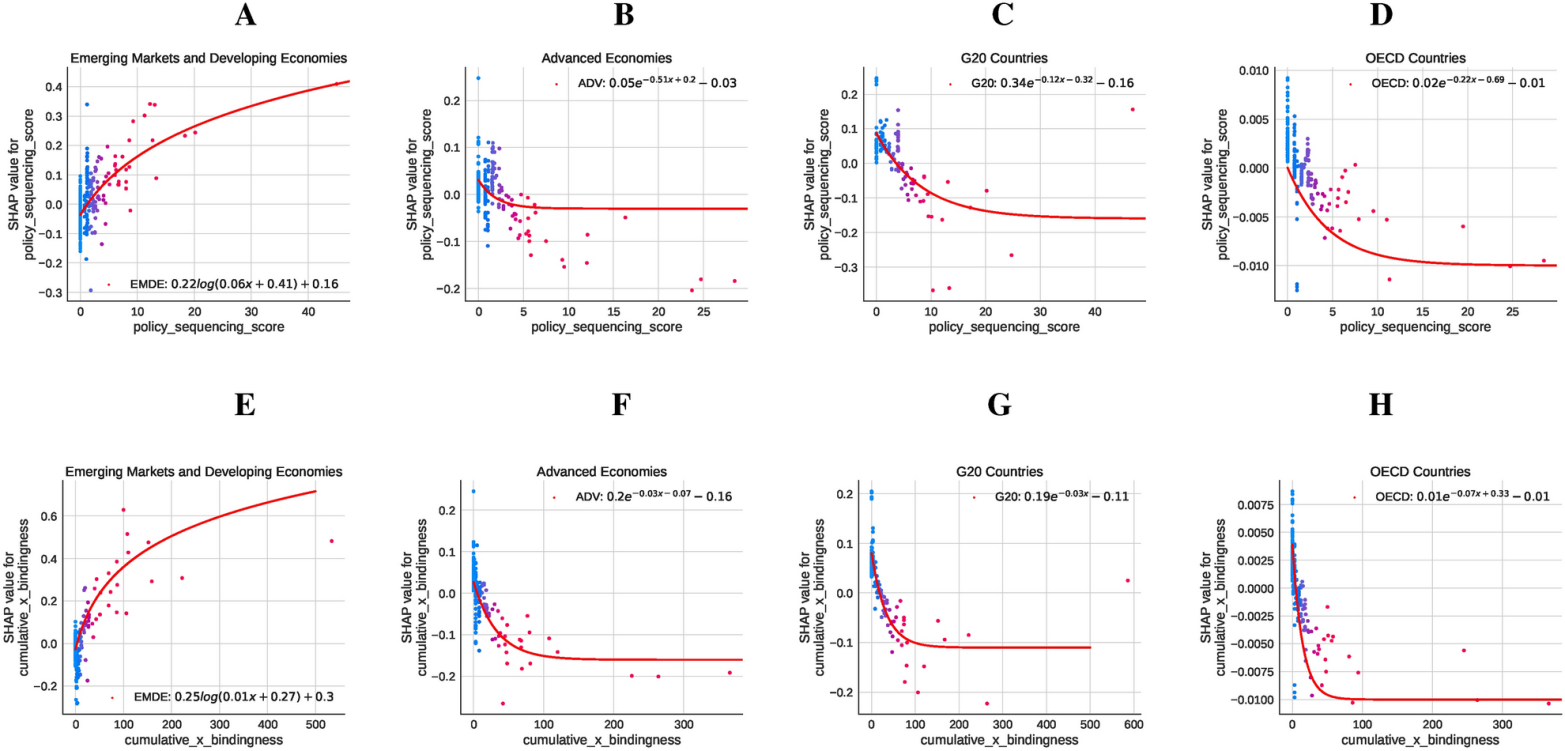
Impact of Policy Sequencing Score (PSS) and Cumulative Adoption with Bindingness on ${{\textbf {CO}}}_2$ Emissions across country groups. Panels (**A** to **D**) (PSS) illustrate the diminishing returns of sequencing: in EMDEs (Panel **A**), the relationship is logarithmic with SHAP values stabilizing at higher scores; Advanced Economies (Panel **B**) show an exponential decay pattern stabilizing around 0.03; G20 countries (Panel **C**) exhibit a similar pattern with stabilization near − 0.01; and OECD countries (Panel **D**) display a flatter exponential relationship, reflecting a minor but consistent role for sequencing. Panels **E** to **H** (Cumulative Adoption with Bindingness) highlight varying impacts of policy enforcement: in EMDEs (Panel **E**), SHAP values rise logarithmically, peaking before stabilizing; Advanced Economies (Panel **F**) show a negative exponential trend stabilizing around − 0.16; G20 countries (Panel **G**) demonstrate an initial increase that converges near − 0.01; and OECD countries (Panel **H**) exhibit a flatter, steady relationship with bindingness converging at approximately − 0.01. These patterns underscore the varying impact of policy measures across economic and institutional contexts.

We also analyze the SHAP values, which quantify the contributions of each feature discussed above to emissions outcomes, revealing distinct trends for each group. Results are shown in Fig. 4.

In EMDEs (Panel A), the fitted curve shows a logarithmic increase, where higher sequencing scores are associated with a positive but diminishing impact on CO2 emissions. This suggests that while sequencing contributes to emissions reductions in other country groups, its effectiveness is hampered in EMDEs, likely due to structural and institutional constraints. Similarly, for CumBind in EMDEs (Panel E), the fitted curve indicates a logarithmic relationship, with higher levels of policy bindingness initially driving steep emissions reductions before plateauing. This highlights the importance of policy adoption and enforcement, even as institutional limitations temper their long-term impact. In Advanced Economies (Panel B), the relationship exhibits exponential decay, where sequencing significantly reduces emissions, stabilizing near a marginal effect of 0.03 at higher scores. Binding policies in these economies (Panel F) display a negative exponential relationship, where emissions are significantly reduced, stabilizing at approximately − 0.16, reflecting the effectiveness of stringent policy implementation. For G20 countries (Panel C), the fitted curve suggests that the impact of sequencing stabilizes at around − 0.01, indicating that beyond a certain point, additional sequencing has minimal incremental effect. A similar pattern is observed for binding policies in G20 countries (Panel G), where SHAP values decline to – 0.01, signaling a sustained but moderate effect of policy enforcement. The trend in OECD countries (Panel D) is relatively flat, stabilizing near − 0.01, suggesting that sequencing plays a consistent yet modest role in emissions reductions. For CumBind in these countries (Panel H), the relationship remains consistently negative, stabilizing near − 0.01. This reflects the modest but reliable contribution of binding policies to emissions reductions within the highly institutionalized settings of OECD economies.

**How policy sequencing and cumulative bindingness shape renewable energy transitions: evidence across institutional contexts**

**Fig. 5:**
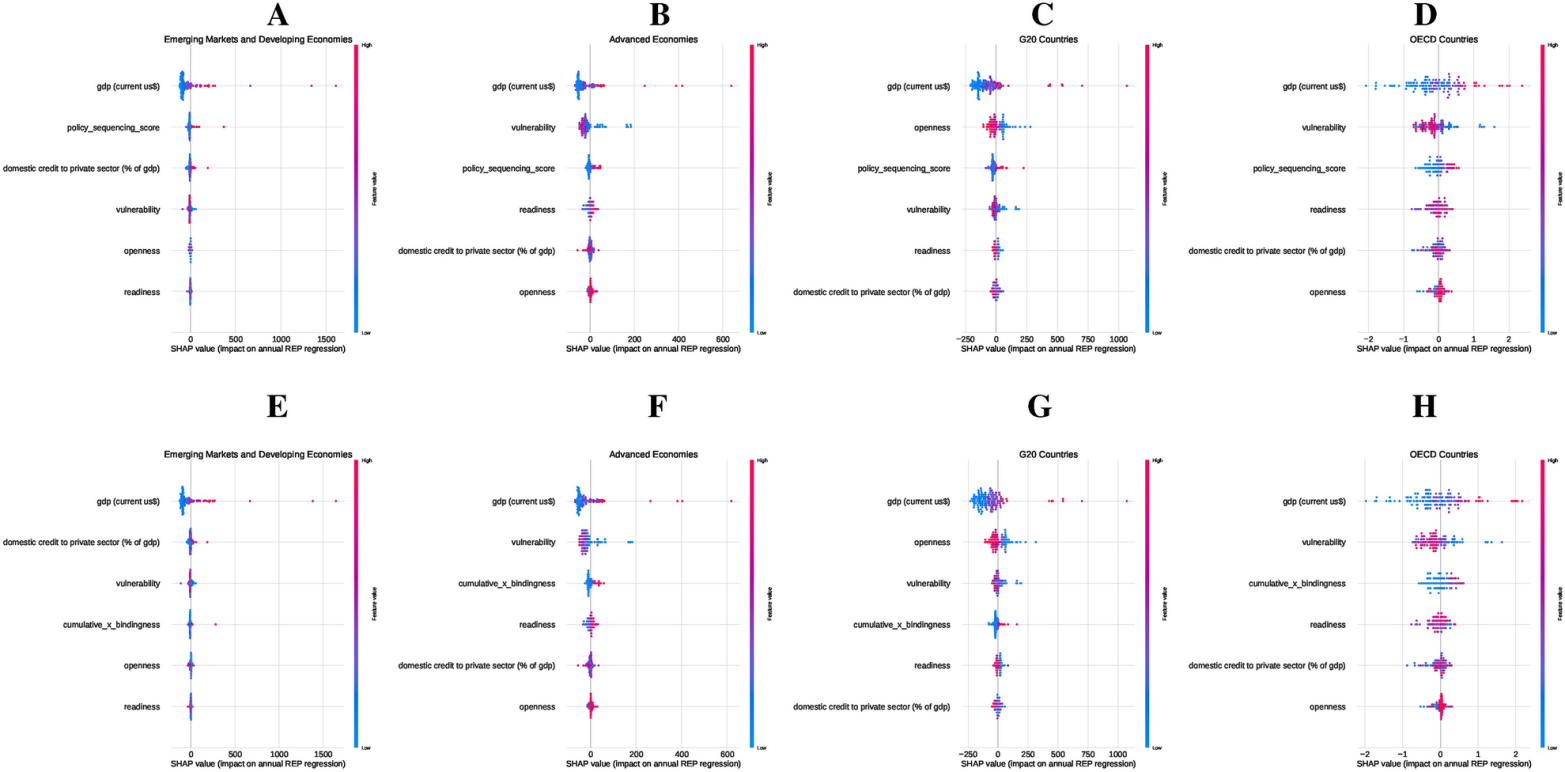
SHAP summary plots illustrating the impact of Policy Sequencing Score (Panels **A**–**D**) and Cumulative Adoption with Bindingness (Panels **E**–**H**) on Renewable Energy Production across country groups. Panel (**A**) (EMDEs) highlights GDP, vulnerability, and PSS as key contributors. Panel (**B**) (Advanced Economies) identifies GDP, openness, and PSS as dominant factors. Panel (**C**) (G20 countries) emphasizes GDP, openness, and PSS score to the private sector, while Panel D (OECD countries) shows GDP, vulnerability, and PSS as leading contributors. In Panels (**E**-**H**), cumulative bindingness plays an increasingly significant role: Panel (**E**) (EMDEs) highlights GDP and domestic credit as dominant, with cumulative bindingness contributing; Panel F (Advanced Economies) shows GDP, vulnerability, and cumulative bindingness as important; Panel (**G**) (G20 countries) reflects GDP, openness, and cumulative bindingness as key; and Panel (**H**) (OECD countries) reveals steady contributions from GDP, vulnerability, and cumulative bindingness. These plots underscore the varying importance of PSS and enforcement across institutional and economic contexts.

In this section, we examine the impact of PSS and CumBind on renewable energy production (REP) across the four country groups, with SHAP summary plots presented in Fig. 5.

For EMDEs (Panel A), GDP, PSS, domestic credit, and vulnerability emerge as key drivers of REP. Structural economic factors like GDP and credit access dominate, while PSS contributes by stabilizing the policy environment. Vulnerability reflects the critical role of climate and economic risks in shaping renewable energy transitions. In Panel E, GDP, domestic credit, and vulnerability remain major drivers, while CumBind plays a meaningful but secondary role. For Advanced Economies (Panel B), GDP, openness, and PSS are the primary contributors to REP. Economic capacity and international integration drive renewable energy growth, with structured policies fostering stability for long-term investments. Vulnerability plays a smaller role, reflecting fewer external constraints, though resilience planning remains important. In Panel F, GDP, vulnerability, and CumBind are significant factors, with strong policy enforcement amplifying renewable energy development within robust institutional frameworks. For G20 countries (Panel C), GDP, openness, and domestic credit dominate REP outcomes, with PSS providing consistent policy frameworks that align stakeholders in these complex economies. Similarly, Panel G highlights GDP, openness, and vulnerability as key drivers, with CumBind enhancing renewable energy outcomes by addressing diverse institutional challenges. For OECD countries (Panel D), GDP, vulnerability, and PSS are the main contributors, emphasizing the link between economic scale and renewable energy growth while highlighting the importance of policy stability within institutionalized contexts. In Panel H, GDP, vulnerability, and CumBind drive REP, demonstrating the importance of rigorous policy enforcement even in advanced renewable energy systems (Fig. 5).

**Fig. 6:**
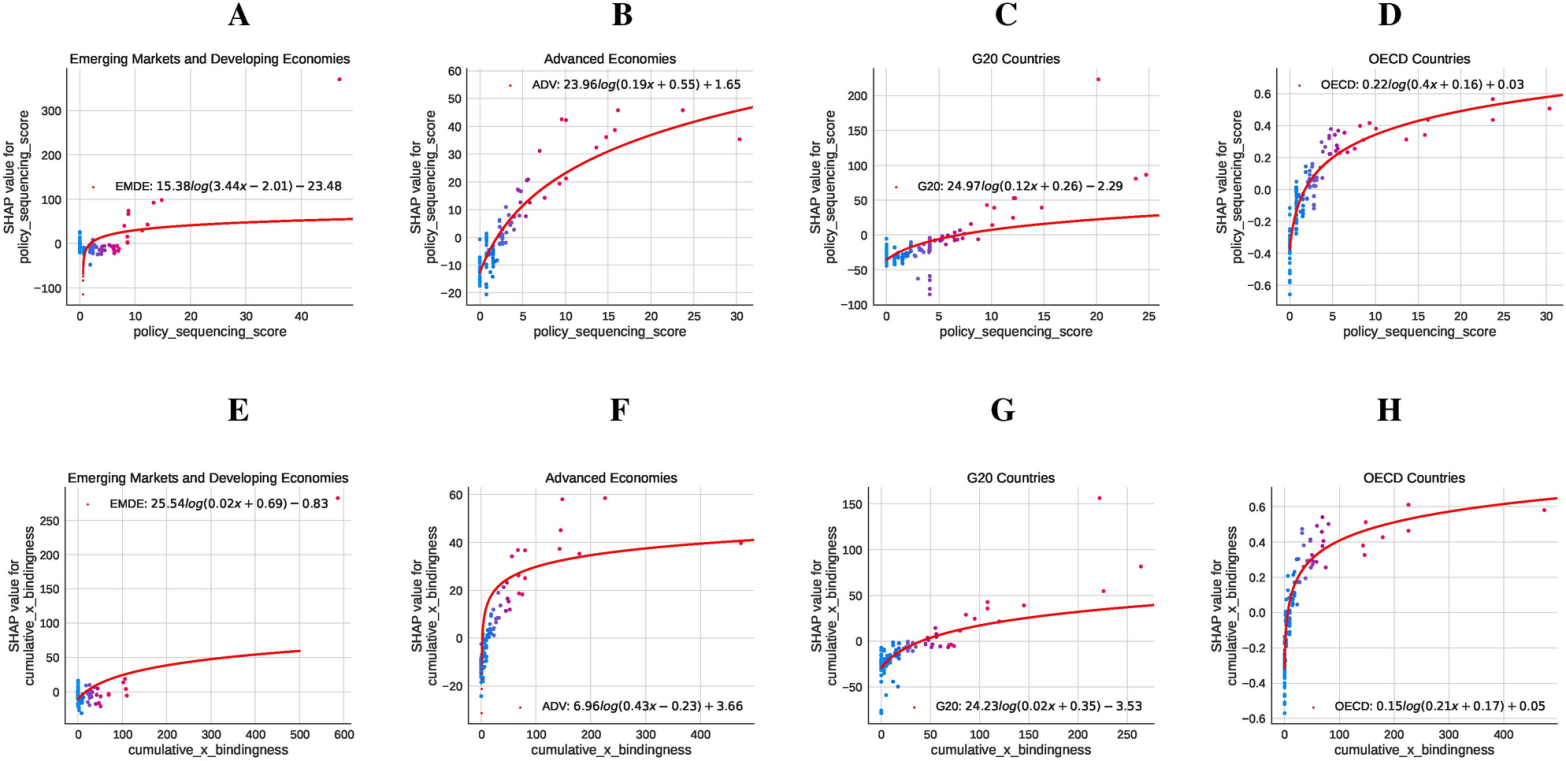
Relationship between Policy Sequencing Score (PSS) and Cumulative Adoption with Bindingness and their contributions to renewable energy production across country groups. SHAP values measure the impact of these policy dimensions, highlighting logarithmic trends with diminishing returns. Panels (**A** and **E**) (EMDEs) show steep initial increases in the contributions of PSS and cumulative bindingness, which stabilize at higher levels due to institutional constraints. Panels (**B** and **F**) (Advanced Economies) indicate more sustained impacts, with diminishing returns reflecting strong institutional frameworks. Panels (**C** and **G**) (G20 countries) exhibit significant initial impacts that level off, reflecting diverse governance structures. Panels (**D** and **H**) (OECD countries) reveal steady but modest contributions consistent with mature policy and energy systems.

We also analyze the SHAP values, which quantify the contributions of each feature discussed above to emissions outcomes, revealing distinct trends for each group. Results are shown in Fig. 6. For EMDEs, PSS (Panel A) shows a logarithmic increase, with sequencing initially contributing significantly to renewable energy production but with diminishing returns at higher levels, likely due to structural and institutional constraints. Similarly, CumBind (Panel E) demonstrates steep initial gains in renewable energy production, followed by stabilization, reflecting the critical role of early policy enforcement even in the face of institutional challenges. In Advanced Economies, PSS (Panel B) exhibits exponential decay, with sequencing driving substantial improvements in renewable energy production before stabilizing at higher scores, indicating reduced marginal returns. CumBind (Panel F) shows a strong and sustained negative relationship with emissions, with significant early reductions stabilizing at higher bindingness levels, underscoring the effectiveness of policy enforcement in institutionalized contexts. For G20 countries, PSS (Panel C) reveals strong initial impacts that taper off, reflecting a consistent framework across diverse governance structures but with limited incremental benefits at higher sequencing levels. Similarly, CumBind (Panel G) shows significant early gains in renewable energy production that stabilize over time, suggesting a moderate but sustained effect of binding policies, even across heterogeneous economies. In OECD countries, PSS (Panel D) presents a flatter trend, with sequencing providing modest but stable contributions to renewable energy production within mature institutional environments. CumBind (Panel H) maintains a consistently negative impact, with steady and reliable support for emissions reductions, reflecting the strong institutional capacity of OECD countries to enforce and sustain binding policies.

### A synthesis of the overall policy impacts across different economic and regional contexts

**Fig. 7:**
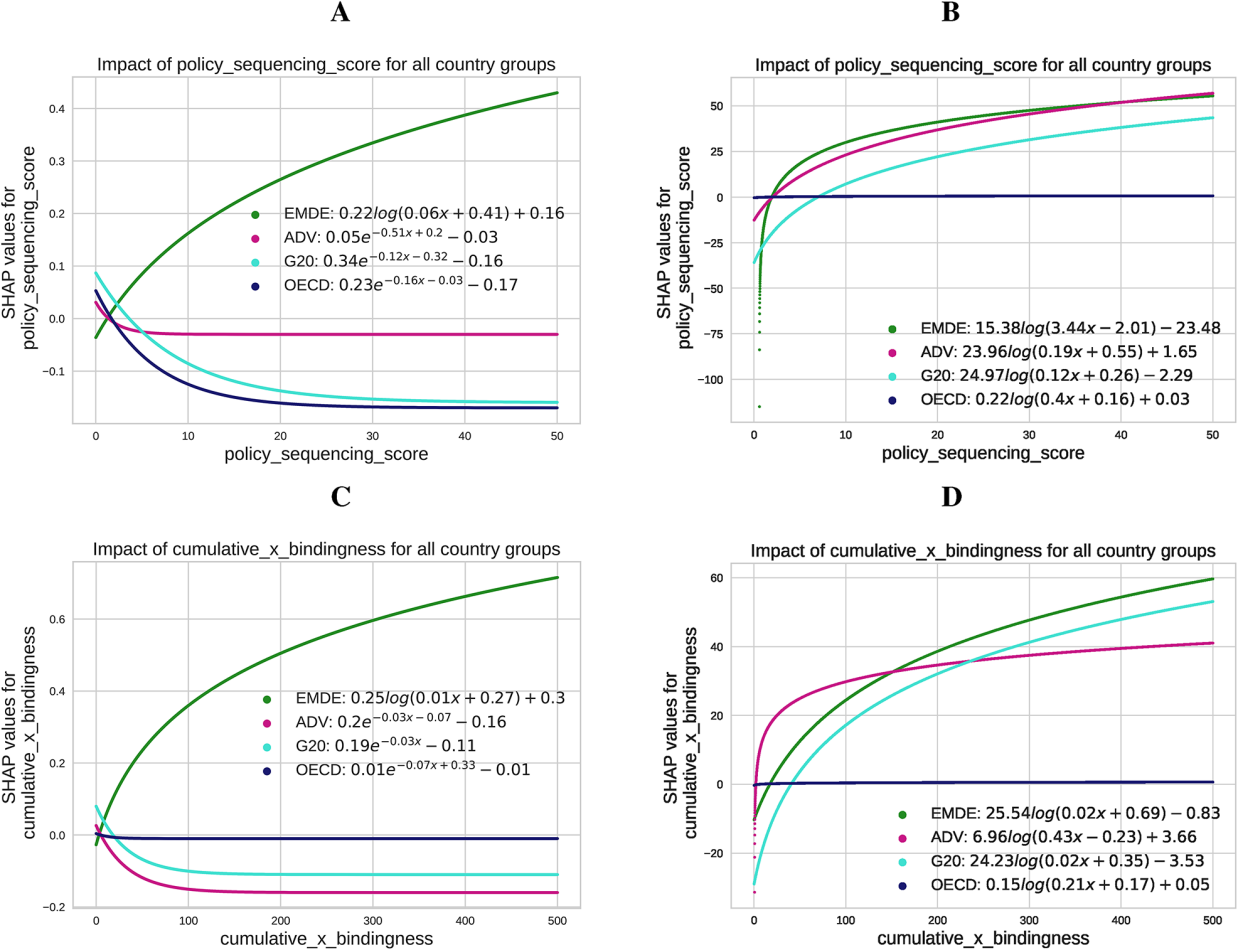
Fitted SHAP values: impact of the policy sequencing score and bindingness-weighted adoption on CO$_2$ emissions (Panels **A** and **C**) and renewable energy production (Panels **B** and **D**) across country groups (EMDE, ADV, G20, OECD). The results highlight differing trends based on structural, institutional, and economic contexts, with notable contrasts between EMDEs and advanced economies.

The analysis and SHAP plot results (Fig. 7) provide a valuable overview of the decarbonization efforts of EMDEs, ADV, G20, and OECD countries and allow for a comprehensive comparison. These findings illustrate how cumulative bindingness-weighted policies and PSS influence emissions (Panels A and C) and REP (Panels B and D), reflecting the structural, institutional, and economic characteristics of each group.

**SHAP analysis of policy sequencing and bindingness across country groups** CumBind policies exhibit varied impacts across country groups. In EMDEs, such policies are associated with short-term increases in emissions due to the prioritization of financial stability over decarbonization and the absence of robust taxonomies and enforcement mechanisms (Panel C). For instance, policies like green bonds and credit allocation measures may channel financial support to carbon-intensive sectors without strict green standards^13,43^. Over time, the SHAP value curves stabilize, indicating reduced sensitivity of emissions to additional cumulative bindingness (Panel C). For renewable energy production, CumBind in EMDEs (Panel D) shows a steep initial association with increased production, but this effect levels off as cumulative policies increase, reflecting the structural and institutional constraints in these economies.

CumBind is associated with lower emissions (Panel C), supported by mature financial systems and institutional structures in advanced economies, including ADV and OECD countries. The declining SHAP values suggest that higher levels of bindingness are increasingly correlated with emissions reductions as policies accumulate. For renewable energy production, advanced economies (Panel D) show flatter SHAP curves, indicating more stable relationships between CumBind and renewable energy outcomes, with diminishing incremental contributions at higher levels of bindingness.

The Policy Sequencing Score (PSS) reveals disparities in how emissions and renewable energy production respond across country groups. In EMDEs, higher PSS levels are linked to limited or even positive correlations with emissions in early stages (Panel A). This reflects challenges like weak enforcement and structural reliance on carbon-intensive industries, which can dilute the impact of sequential measures. Over time, the SHAP values flatten, suggesting diminishing marginal impacts of further sequencing as structural barriers persist. For renewable energy production (Panel B), PSS shows initial positive associations that taper off at higher levels, consistent with the limitations posed by institutional and economic challenges.

In advanced economies, PSS is consistently associated with lower emissions across its range (Panel A). The SHAP values highlight a more predictable relationship, as sequencing advances alongside decarbonization pathways. For renewable energy production (Panel B), PSS shows stable and positive associations with increased renewable output, reflecting the structured environments in which these policies are applied.

For G20 countries, CumBind (Panel C) displays early associations with reduced emissions that stabilize over time, likely reflecting the diversity of institutional capacities within the group. For renewable energy production (Panel D), the relationship with CumBind is positive but less pronounced, as the institutional heterogeneity within the G20 moderates the relationship. PSS in G20 countries (Panel A) shows consistent associations with reduced emissions, though the relationship flattens at higher sequencing levels. For renewable energy (Panel B), PSS shows moderate gains, particularly in advanced economies within the group, while emerging economies display more varied outcomes.

In OECD countries, CumBind (Panel C) is associated with consistently lower emissions, with SHAP values stabilizing as bindingness increases. For renewable energy production (Panel D), CumBind exhibits a relatively flat relationship, reflecting limited variation as renewable energy integration becomes saturated. PSS (Panel A) shows steady negative correlations with emissions, with SHAP curves flattening at higher sequencing levels. For renewable energy production (Panel B), PSS is positively associated with stable and predictable increases in renewable energy, aligning with the institutional capacity and advanced policy frameworks of OECD countries.

**Fig. 8:**
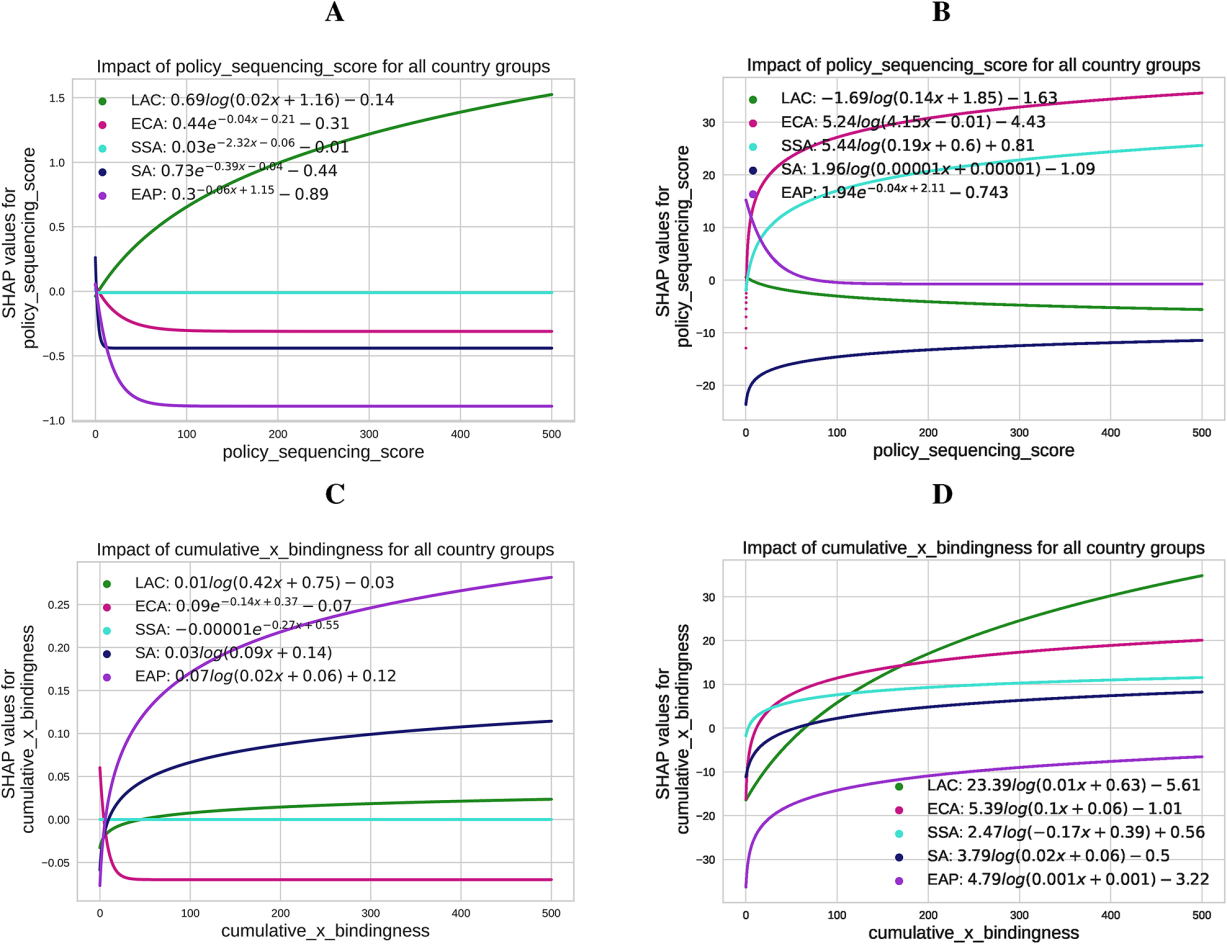
Fitted SHAP values illustrating the impact of the policy sequencing score and the bindingness-weighted adoption on CO$_2$ emissions and renewable energy production across different country groups. Panel (**A**) displays the SHAP values for the effect of the PSS score on $\hbox {CO}_2$ emissions for regions: Latin America and the Caribbean (LAC), Europe and Central Asia (ECA), Sub-Saharan Africa (SSA), South Asia (SA), and East Asia and the Pacific (EAP). Panel (**B**) shows the SHAP values for the PSS score’s impact on renewable energy production for the same regions. Panels C and D depict the SHAP values for the effect of cumulative bindingness-weighted policy adoption on CO$_2$ emissions (Panel **C**) and renewable energy production (Panel **D**) across these regions. The fitted SHAP values are calculated using logarithmic transformations, capturing region-specific variations in the influence of policy adoption and sequencing.

**Regional variations in the influence of policy sequencing and bindingness on CO2 emissions and renewable energy production** Finally, we analyze regional differences in the impact of PSS and bindingness-weighted adoption on CO2 emissions and REP, as shown in Fig. 8. Panels A and B highlight the influence of the PSS, which varies across regions in both magnitude and functional form.

In Panel A, East Asia and the Pacific (EAP) shows the steepest decline in SHAP values for CO2 emissions as PSS increases, indicating a strong relationship between sequencing and emissions reductions. This may reflect the region’s lower initial baseline for policy implementation, where small improvements in PSS correspond to notable changes in emissions. By contrast, Latin America and the Caribbean (LAC) display a steep increase. Panel B reveals a nonlinear positive relationship between PSS and REP in Sub-Saharan Africa (SSA), suggesting that renewable energy production is highly responsive to sequencing improvements. In East Asia and the Pacific (EAP), the trend is flatter, reflecting a less pronounced relationship, potentially due to the region’s advanced renewable energy sector, where additional policies yield diminishing marginal effects.

Panels C and D examine the effects of CumBind. Panel C shows a positive relationship between CumBind and CO2 emissions in EAP, South Asia (SA), and LAC, as reflected by increased SHAP values. This suggests that binding policies correlate with higher emissions in these regions. In contrast, Europe and Central Asia (ECA) display a downward trend, indicating a less pronounced relationship, which may reflect diminishing returns in regions with established policy landscapes. Panel D illustrates the strongest positive relationship between CumBind and REP in the regions of LAC, SSA, and ECA, as indicated by a sharp increase in SHAP values. This suggests that these regions effectively leverage binding policies to expand renewable energy, likely driven by robust technical expertise and financial capacity. In SSA, however, the increase is more gradual, which may reflect a smaller market size and slower technology diffusion.

**Addressing climate risks through policies** The analysis highlights significant regional and country-group differences in how climate-related financial policies relate to CO2 emissions and REP. Emerging markets and developing economies (EMDEs) face pronounced physical risks, such as extreme weather events and resource scarcity, alongside structural reliance on carbon-intensive sectors^44^. Transition risks, including stranded assets and economic disruptions from stricter global climate policies, also present challenges^45^. In these contexts, Sub-Saharan Africa (SSA) demonstrates a nonlinear positive relationship between PSS and REP, indicating that renewable energy production is highly responsive to improvements in sequencing. However, the moderate increase reflects a still small market and slower technology diffusion. Conversely, SSA shows a less pronounced relationship between CumBind and CO2 emissions, underscoring the ongoing challenges in leveraging binding policies to reduce emissions.

South Asia (SA) exhibits a positive relationship between CumBind and CO2 emissions, as SHAP values indicate that binding policies correlate with higher regional emissions. This finding suggests potential implementation gaps or the difficulty of addressing entrenched reliance on carbon-intensive sectors.

Latin America and the Caribbean (LAC) reveal contrasting dynamics. While PSS correlates with increased CO2 emissions, suggesting challenges in effective sequencing, CumBind shows a strong positive relationship with REP. This highlights the region’s ability to use binding policies to expand renewable energy, supported by technical expertise and financial capacity. The steep trends underscore the region’s responsiveness to these policies, though CO2 reductions remain elusive.

East Asia and the Pacific (EAP) shows a steep decline in SHAP values for CO2 emissions with increases in PSS, reflecting a strong relationship between sequencing and emissions reductions. This may result from the region’s lower initial baseline for policy implementation, where incremental improvements yield significant gains. However, for REP, EAP displays a flatter relationship with both PSS and CumBind, likely due to its advanced renewable energy sector, where additional policies have diminishing marginal effects.

In Europe and Central Asia (ECA), CumBind shows a downward trend in its relationship with CO2 emissions, likely reflecting diminishing returns in regions with well-established policy landscapes. However, CumBind has a strong positive relationship with REP, emphasizing the potential for binding policies to further renewable energy expansion, even in regions with more mature markets.

In advanced economies and OECD countries, lower physical vulnerabilities and established regulatory frameworks contribute to consistent decarbonization trajectories. However, flatter trends in SHAP results for REP and CO2 emissions indicate diminishing sensitivity to additional policies, reflecting policy saturation and existing infrastructure’s limitations for further improvements.

The G20 countries present a dual reality: advanced economies follow patterns observed in OECD countries, with stable emissions reductions and REP growth, while emerging economies display trends similar to EMDEs. This divergence highlights the varying capacities and challenges within the G20, as some countries benefit from established frameworks while others face institutional and financial constraints.

## Conclusions

This study underscores the critical role of Climate-Related Financial Policies (CRFPs) in aligning financial systems with decarbonization goals. By analyzing the relationships between Policy Sequencing Scores (PSS), cumulative bindingness (CumBind), and key environmental indicators, the findings reveal significant regional and country-group differences in how these policies relate to CO2 emissions and renewable energy production (REP). A summary of these trends, challenges, and corresponding policy recommendations across regions is provided in Table 1.

**Table 1:** Observed trends in CRFP impacts across country groups and regions.

Group/region	Observed impacts of CRFPs	Key challenges	Policy recommendations
Advanced Economies (ADV)	Consistent reductions in $\hbox {CO}_2$ emissions and long-term REP growth, with diminishing marginal impacts of additional policies due to advanced infrastructure	Stable institutional frameworks but diminishing returns on additional policies	Prioritize innovation and cross-border collaboration to address diminishing returns and enhance decarbonization strategies
OECD Countries	Steady emissions reductions and REP growth, supported by mature institutional frameworks and coherent policy implementation	Established policy landscapes with diminishing marginal impacts at higher levels of policy adoption	Enhance collaboration and technological diffusion to sustain progress in emissions reduction and renewable energy expansion
Emerging Markets and Developing Economies (EMDEs)	Mixed impacts; strong responsiveness to binding policies in specific regions (e.g., SSA, LAC), but limited effectiveness in reducing CO$_2$ emissions in others	Institutional and structural barriers, including reliance on carbon-intensive industries and limited enforcement mechanisms	Strengthen international cooperation, capacity building, and access to green finance to address institutional and economic barriers
G20 Countries	Advanced economies show stronger trends, while emerging economies face challenges with policy coherence and enforcement	Wide variation in institutional capacity and economic structure	Tailor strategies to country-specific contexts, focusing on building institutional capacity and aligning policies across diverse economies
Sub-Saharan Africa (SSA)	Moderate relationship between bindingness-weighted policies and $\hbox {CO}_2$ emissions; nonlinear positive impact on REP growth	High vulnerability, institutional constraints, and limited renewable energy capacity	Increase international funding, capacity building, and technical assistance to scale impacts and unlock renewable energy potential
South Asia (SA)	Binding policies correlate with higher $\hbox {CO}_2$ emissions; REP growth shows mixed responsiveness to sequencing improvements	Pronounced physical risks and limited institutional readiness	Address institutional inefficiencies and scale up investment frameworks to maximize gains
East Asia and the Pacific (EAP)	Strong relationship between sequencing and $\hbox {CO}_2$ emissions reductions; flatter REP growth trends due to diminishing returns	Advanced renewable energy sector with diminishing marginal impacts of additional policies	Focus on optimizing policies to sustain growth and mitigate diminishing returns
Latin America and the Caribbean (LAC)	Sequencing policies correlate with increased $\hbox {CO}_2$ emissions; bindingness-weighted policies strongly drive REP growth	Limited responsiveness to sequencing improvements and enforcement gaps	Strengthen enforcement mechanisms and accelerate the adoption of advanced policy instruments
Europe and Central Asia (ECA)	Positive relationship between binding policies and REP growth; limited incremental impacts on CO$_2$ emissions due to policy saturation	Policy saturation and weaker responsiveness to additional policies due to slower technology diffusion	Encourage policy innovation, expand green market opportunities, and foster technology adoption

The results highlight the strong associations between PSS and CumBind with decarbonization outcomes, particularly in Emerging Markets and Developing Economies (EMDEs). Sub-Saharan Africa (SSA) and South Asia (SA) show the steepest relationships between these policies, emissions reductions, and renewable energy growth, reflecting untapped potential and responsiveness to policy sequencing and enforcement. However, structural and institutional constraints, such as limited financial capacity and weaker enforcement mechanisms, moderate these relationships and emphasize the need for targeted international support to unlock further progress.

Latin America and the Caribbean (LAC) exhibit more gradual relationships between PSS, CumBind, and environmental outcomes. While PSS shows an initial positive correlation with REP, the relationship flattens at higher levels, suggesting slower responsiveness compared to other regions. Similarly, the cumulative impact of bindingness-weighted policies shows weaker associations with emissions reductions and renewable energy growth, reflecting the region’s more mature policy frameworks and incremental gains.

Advanced economies and OECD countries demonstrate stable relationships between PSS, CumBind, and environmental outcomes, supported by robust institutional frameworks and predictable policy environments. SHAP results reveal consistent emissions reductions and REP growth, though diminishing sensitivity to additional policies suggests the need to focus on innovation and cross-sectoral integration to sustain progress. For example, East Asia and the Pacific (EAP) benefits from its advanced renewable energy sector, while Europe and Central Asia (ECA) show weaker responses, reflecting policy saturation and slower technology diffusion.

The G20 presents a dual dynamic, with advanced economies exhibiting trends similar to OECD countries and emerging economies reflecting patterns observed in EMDEs. This divergence highlights the heterogeneity of institutional and economic capacities within the group, as some countries benefit from established frameworks while others face significant barriers to policy implementation and enforcement.

Overall, these findings emphasize the importance of tailoring climate-related financial policies to specific economic and institutional contexts. As summarized in Table 1, sequencing foundational policies to build stable environments, enhancing enforceability through mandatory measures, and strengthening international collaboration will be critical to aligning financial systems with global climate objectives. The study concludes that achieving meaningful progress in emissions reductions and renewable energy expansion requires a strategic combination of policy innovation, institutional strengthening, and coordinated international efforts to accelerate the transition to a low-carbon economy and enhance climate resilience.

Future research should focus on the synergies and interactions between Climate-Related Financial Policies (CRFPs) and complementary policy domains, including environmental, fiscal, and trade frameworks, to comprehensively assess their collective impact on decarbonization and renewable energy transitions. A critical area of investigation lies in the interplay between policy sequencing and market-based mechanisms, such as carbon pricing and renewable energy subsidies, to evaluate the efficacy of coordinated policy strategies in driving climate action. Moreover, the scope of analysis should be broadened to integrate additional sustainability dimensions, including biodiversity preservation, nature-based solutions, and circular economy principles, which are inherently linked to achieving overarching climate objectives. Finally, future studies should examine the equity implications of CRFPs, analyzing their social and economic effects across diverse demographic groups to ensure that policy outcomes are not only effective but also equitable and inclusive, fostering a just transition to a low-carbon economy.

## Methods

### Data

The data used in the analysis are summarized in Table 2 and described in detail below.

**Table 2:** Data: description and sources.

Acronym	Data definition	Data description	Source
GPP	Green Prudential Regulations	Policies aimed at identifying and mitigating climate-related financial risks to ensure financial stability	Climate-related financial policy database^26^
GCA	Green Credit Allocation Policies	Policies encouraging green lending and investments through credit allocation and lending limits	Climate-related financial policy database^26^
GFG	Green Financial Guidelines	Policies supporting the development of green or climate-aligned financial markets	Climate-related financial policy database^26^
OGD	Other Disclosure Requirements	Policies encouraging public disclosure of climate-related financial risks, including for non-financial institutions	Climate-related financial policy database^26^
GB	Green Bonds	Policies promoting green lending through green bonds, including taxonomy and issuing	Climate-related financial policy database^26^
$\hbox {CO}_2$ Emissions	$\hbox {CO}_2$ Emissions	Measures each country’s contribution to greenhouse gas emissions over time	Our World in Data^46^
Renewable Energy Production	Renewable Energy Capacity per Capita	Total renewable energy capacity per capita, measured in watts, covering multiple renewable sources	IEA Renewables Data Explorer
Readiness	Climate Resilience Readiness Index	Measures a country’s readiness to leverage climate finance and adaptation opportunities	ND-GAIN database^47^
Vulnerability	Climate Vulnerability Index	Assesses a country’s vulnerability to climate risks	ND-GAIN database^47^
Chinn-Ito	Financial Openness Index	Measures the degree of financial openness	Chinn-Ito Index
GDP	Gross Domestic Product	Represents economic performance, measured in US dollars	World Bank
Domestic Credit	Domestic Credit to Private Sector	Percentage of GDP representing the availability of credit in the private sector	World Bank World Development Indicators

### Sample

**Fig. 9:**
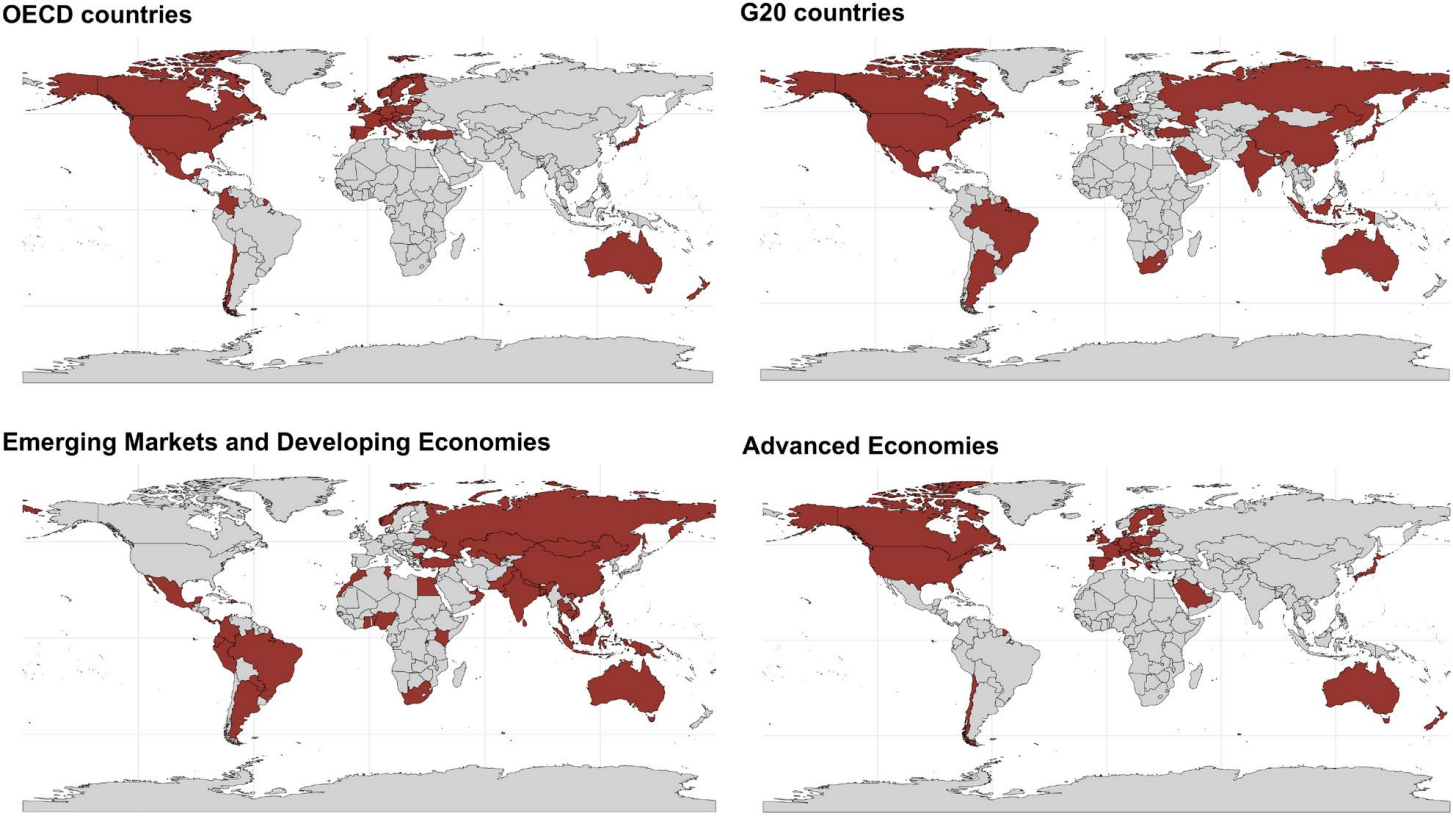
Country coverage by group. Maps showing the geographical distribution of countries classified as OECD members, G20 countries, Emerging Markets and Developing Economies (EMDEs), and Advanced Economies. Highlighted regions (in red) indicate the countries included in each group, illustrating the diverse global representation underlying the analysis.

The study includes data from both developed and developing economies to capture the heterogeneity in Climate-Related Financial Policies (CRFPs) implementation and outcomes. Developed economies are characterized by established institutional frameworks and extensive policy histories, providing insights into mature approaches. In contrast, developing economies illustrate how structural constraints, institutional capacity, and resource limitations influence policy adoption and effectiveness. This diverse sample enables a balanced and nuanced analysis of global patterns and regional differences in CRFP impacts. The sample comprises 87 countries, of which 34 are OECD countries, 20 are G20 countries, 39 are advanced economies (ADV), and 48 are classified as emerging markets and developing economies (EMDEs). An overview of the distribution of countries across the groups is provided in Fig. 9.

For the regional analysis, we rely on the World Bank region classification, according to which regions are composed as follows:

- The **East Asia and Pacific (EAP)** region includes China, Mongolia, South Korea, Japan, Malaysia, Indonesia, Philippines, Vietnam, Thailand, Papua New Guinea, Fiji, Cambodia, Australia, New Zealand, Singapore, Timor-Leste, and Korea.
- The **Europe and Central Asia (ECA)** region includes France, Germany, Italy, Spain, United Kingdom, Poland, Turkey, Ukraine, Kazakhstan, Russia, Georgia, Armenia, Austria, Belgium, Bulgaria, Croatia, Cyprus, Czechia, Denmark, Estonia, Finland, Greece, Hungary, Iceland, Ireland, Latvia, Lithuania, Luxembourg, Malta, Netherlands, Norway, Portugal, Romania, Slovak Republic, Slovenia, Sweden, and Switzerland.
- The **Latin America and the Caribbean (LAC)** region includes Brazil, Argentina, Chile, Mexico, Colombia, Peru, Venezuela, Panama, Costa Rica, Ecuador, Paraguay, Dominican Republic, Trinidad & Tobago, Cuba, Honduras, Jamaica, Nicaragua, and Haiti.
- The **South Asia (SA)** region includes India, Pakistan, Sri Lanka, Bangladesh, Nepal, Bhutan, Maldives, and Afghanistan.
- The **Sub-Saharan Africa (SSA)** region includes South Africa, Nigeria, Ethiopia, Kenya, Ghana, Angola, Benin, Botswana, Tanzania, Zambia, Uganda, Zimbabwe, Seychelles, and Rwanda.

#### Climate-related financial policies data

We used an updated version of the climate-related financial policy database^26^, which originally included data up to 2020. Data from 2021 to 2023 were collected manually using a structured, multi-step approach to ensure comprehensive coverage of climate-related financial policies. First, a taxonomy was used to categorize policies into five main areas: green prudential regulations, green credit allocation policies, green financial guidelines, green disclosure requirements, and green bonds. We systematically searched official documents on the websites and databases of central banks, financial regulators, ministries, and banking associations using targeted keywords related to climate finance and regulation. Each policy was classified by bindingness (mandatory, voluntary, or non-binding), and the responsible authorities were noted. Documents were then read, validated, and cross-checked to ensure accuracy and avoid duplication. Full methodological details are available in^5^ and^26^.

The newly created dataset covers 87 countries, focusing on G20 and OECD countries. To categorize the policy types considered in our study, we relied on the taxonomy of the five policy areas comprising the climate-related financial policy index (CRFPI)^5^. Accordingly, the investigation considers policies in the financial sector that have the following objectives:

- Identifying and protecting against climate-related financial risks to ensure financial stability, known as *green prudential regulations (GPP)*.
- Encouraging green lending and investments using methods like credit allocation and lending limits, referred to as *green credit allocation policies (GCA)*.
- Supporting the development of green or climate-aligned financial markets, referred to as *green financial principles (GFG)*.
- Encouraging public disclosure of climate-related financial risks, including disclosure requirements for non-financial institutions such as insurance companies and pension funds, referred to as *other disclosure requirements (OGD)*.
- Promoting green lending through green bonds, which involves *green bonds taxonomy and issuing (GB)*.

#### Environmental indicators

${{\textbf {CO}}}_2$ **emissions** Data on CO$_2$ emissions, sourced from Our World in Data’s CO$_2$ and Greenhouse Gas Emissions database^46^, play a central role in this study by measuring each country’s contribution to greenhouse gas emissions over time. This dataset offers a detailed account of CO$_2$ emissions from various sectors, enabling an in-depth analysis of trends in emissions levels and their relation to climate-related financial policies. By tracking changes in emissions alongside the adoption of specific financial policies, the study aims to evaluate the impact of these policies in reducing carbon emissions. This data source, being publicly accessible and regularly updated, supports robust cross-country comparisons and allows for longitudinal analysis, contributing valuable insights into the potential role of financial regulations and incentives in mitigating climate change.

**Renewable energy production** The second environmental indicator is the total renewable energy capacity per capita in watts sourced from the IEA Renewables Data Explorer. This measure encompasses the combined capacity of various renewable energy sources, including bioenergy, hydropower (which also takes into account pumped storage capacity), onshore and offshore wind energy, solar photovoltaics (PV), concentrated solar power (CSP), geothermal energy, and ocean-based energy technologies. This is a crucial measure for understanding the extent of renewable energy adoption. The variable “Renewable Energy Production” includes data for 37 unique countries in the dataset, with the following group coverage: 18 countries classified as Advanced Economies, 20 as Emerging Markets and Developing Economies (EMDEs), 19 as OECD countries, and 20 as G20 countries.

**Other variables** The analysis incorporates readiness and vulnerability indices from the ND-GAIN database, which assesses each country’s climate resilience. Additionally, we include the Chinn-Ito index, a measure of financial openness, to explore how open financial systems may interact with climate-related financial policies. GDP data from the World Bank represent economic performance, while domestic credit to the private sector as a percentage of GDP is used as a financial indicator, reflecting credit availability.

#### Diagnostics on data

**Fig. 10:**
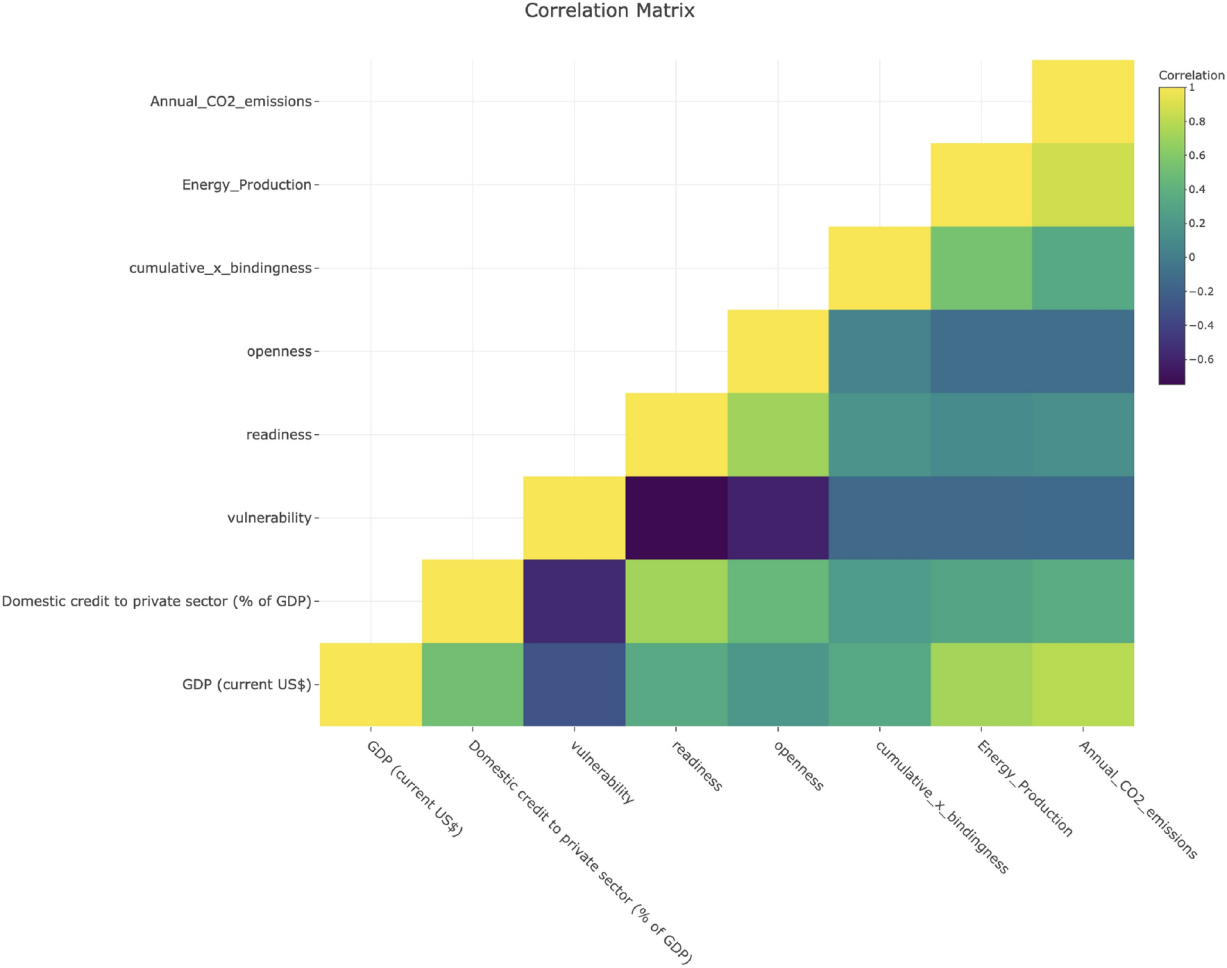
Correlation matrix.

The correlation matrix in Fig. 10 shows key relationships among the selected variables. Notably, a moderate positive correlation between Annual CO$_2$ Emissions and Energy Production suggests a link between energy production activities and CO$_2$ emissions. Additionally, Domestic Credit to the Private Sector (% of GDP) exhibits weak or negligible correlations with variables such as Readiness and Openness, indicating limited linear associations between these factors. The matrix also highlights some negative correlations, which could indicate potential trade-offs or inverse relationships between certain variables.

#### Policy length and bindingness

Using the data on climate-related financial policies, we compute two policy measures. The *Cumulative Policy Index* measures the cumulative number of policies adopted by a country over time, reflecting the total policy activity related to climate or other relevant areas. It is computed by summing up all policy adoptions for each country from the beginning of the observed period up to a given year. For example, if a country adopted two policies in 2000 and three more in 2005, its cumulative policy index in 2005 would be five.

The *Cumulative Policy Index* $\times$ *Bindingness* incorporates the concept of policy “bindingness” to reflect the relative strength or enforceability of policies. Each policy is assigned a weight based on its bindingness (e.g., non-binding, voluntary, or mandatory). The cumulative index is then calculated by summing the binding-weighted policies adopted over time. In the case of multiple policy adoptions in a given year, the index considers the sum of all adopted policies and their respective bindingness scores for that year. This metric captures the number of policies adopted and their enforceability, providing more detailed information on a country’s policy efforts.

#### Policy sequencing score: computation method

The Policy Sequencing Score is a measure designed to evaluate the alignment of a country’s policy adoption patterns with typical global sequences. It is calculated in two main steps:

**Step 1: Conditional Frequencies of Policy Adoptions.** First, all unique policies (*Policy1, Policy2, ...*) are identified in the dataset. For every pair of policies (*Policy1* and *Policy2*), the conditional frequency is computed. This frequency represents how often *Policy1* is adopted before *Policy2*, normalized by the total number of times *Policy2* is adopted. Mathematically, the conditional frequency is calculated as:

$$\text {Frequency} = \frac{\text {Number of times Policy1 precedes Policy2}}{\text {Total number of times Policy2 is adopted}}$$

**Step 2: Computation of Policy Sequencing Score.** For each country and year, the Policy Sequencing Score is calculated by summing the conditional frequencies of the policies adopted by that country up to and including the given year. The process involves:

1. Filtering all policies adopted by a country up to the specified year.
2. Summing the conditional frequencies associated with these policies. If a policy’s frequency is missing, it is treated as zero. Formally, the Policy Sequencing Score is computed as:
  $$\text {Policy Sequencing Score} = \sum _{i=1}^{n} \text {Conditional Frequency of Policy}_i$$
  where $n$ is the total number of policies adopted by the country up to the specified year.

### Methodology

#### Feature selection via classical Machine Learning techniques

Machine learning offers various methodologies for applications such as regression, classification, and complex decision-making processes. This study focuses on Classical Machine Learning algorithms, deliberately setting aside the expansive field of Deep Learning to concentrate on what is commonly referred to as Shallow Machine Learning. We aim to analyze the impact of policy sequence implementations on two key variables critical to the low-carbon transition: emissions metrics and clean energy generation. This analysis aims to uncover the intricate dynamics that govern the interaction between policy initiatives and progress toward a sustainable, carbon-constrained future. Our methodology involves systematically examining classic machine learning techniques to determine the most effective method for variable selection. By identifying each variable’s specific influence within our selected subset, we seek to refine our understanding of their individual and collective contributions to the dependent variables under study.

#### Algorithmic selection from Classical Machine Learning collection

To ensure precise effect measurement, our study adopts a comprehensive approach by evaluating 19 different classical machine learning regressors available in PyCaret. PyCaret^48^ is an open-source low-code machine learning library in Python that streamlines the training process and comparing models. It provides a unified interface for a wide range of algorithms and automates repetitive tasks, making it an efficient tool for experimentation and analysis. The performance of these regressors is assessed using six key metrics: Mean Absolute Error (MAE), Mean Squared Error (MSE), Root Mean Squared Error (RMSE), Coefficient of Determination ($R^2$), Root Mean Squared Logarithmic Error (RMSLE), and Mean Absolute Percentage Error (MAPE). We deploy these regressors to model two dependent variables-emissions and clean energy production-based on various policy sequence lengths while incorporating control variables. Among the models evaluated, the Extra Trees Regressor outperforms the others across all six metrics and is therefore selected as the primary regressor for this investigation.

#### Extra-trees regressor: methodology and interpretable feature selection

**Fig. 11:**
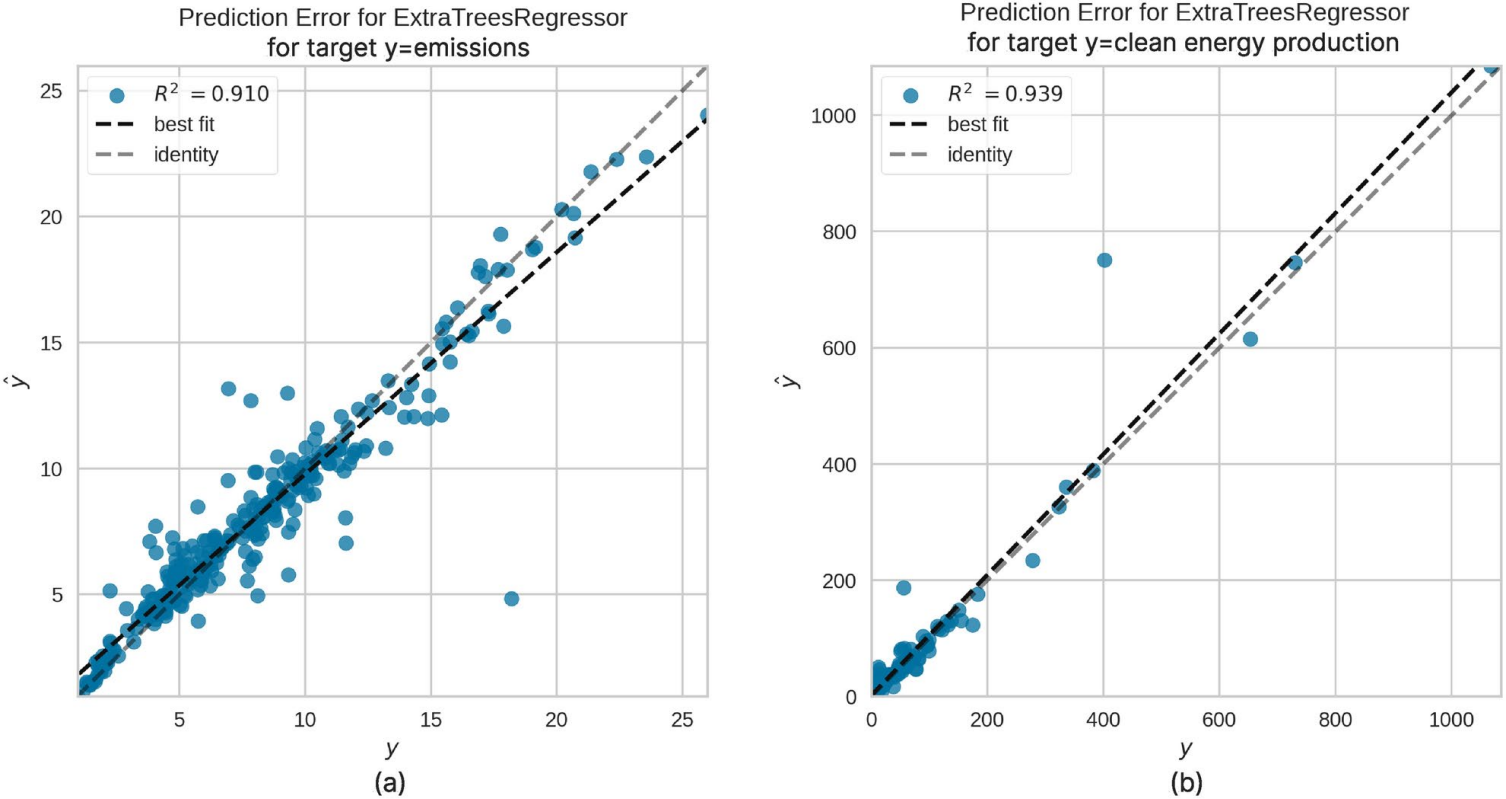
An example of fitting results of ExtraTrees Regression model for the targets (**a**) emissions and (**b**) clean energy production.

We selected the Extra Trees Regressor^49^ for our analysis due to its effectiveness in handling noisy and high-dimensional economic data. This ensemble learning method enhances traditional decision trees by introducing additional randomness: at each decision node, it selects random subsets of features and random splitting thresholds. This approach results in a diverse collection of trees, improving the model’s robustness and reducing the risk of overfitting. The algorithm’s ability to explore a wide range of decision pathways allows it to capture complex patterns and identify influential variables that might be overlooked in more deterministic models. The Extra Trees Regressor demonstrated high accuracy on a validation set comprising $30\%$ of our dataset, with $R^2$ values exceeding 0.9 for emissions and clean energy production, as demonstrated in Fig. 11. This indicates that the model provides reliable and credible insights into the factors influencing these key dependent variables. The Extra Trees Regressor configuration for this analysis is specified as follows:


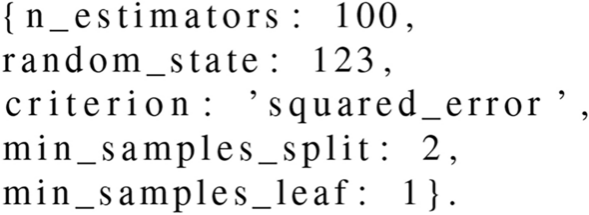

#### Detailed exploration of the impact of variables using Classical Machine Learning and SHapley Additive exPlacountries (SHAP) values

This investigation advances by analyzing the effects of variables using classical machine learning models, supplemented by SHapley Additive exPlanation (SHAP) values, to achieve a refined understanding of feature impacts.

#### Motivation for using Shapley values

While the Extra Trees Regressor offers an initial assessment of feature importance, it does not provide detailed insights into how changes in individual control or independent variables quantitatively affect the dependent variable’s predictions. This limitation stems from its aggregate measure of importance, which lacks the granularity needed to understand the specific impact of each variable. To address this gap, we employ Shapley values^50^, a concept from cooperative game theory adapted for machine learning interpretability through SHAP (SHapley Additive exPlanations)^38^. Shapley values offer a theoretically sound method for attributing a model’s prediction to its input features by considering all possible subsets of features and their contributions.

Mathematically, the Shapley value for a feature *i* in a model *f* is defined as:

1 $$\begin{aligned} \phi _i(f) = \sum _{S \subseteq N \setminus {i}} \frac{|S|!(n - |S| - 1)!}{n!} \left[ f(S \cup {i}) - f(S) \right] , \end{aligned}$$

where:

- *N* is the set of all features,
- *S* is any subset of *N* excluding feature *i*,
- *n* is the total number of features,
- *f*(*S*) represents the model’s prediction using the features in subset *S*,
- The term $\frac{|S|!(n - |S| - 1)!}{n!}$ ensures a fair weighting across all subsets.

By calculating the average marginal contribution of feature *i* across all possible combinations of features, Shapley values provide a nuanced and equitable quantification of each feature’s impact on the model’s predictions. This method enhances interpretability by revealing which variables are important and how they influence the dependent variable. Such detailed insights are essential for understanding the complex interactions in our economic models and for making informed policy decisions regarding emissions and clean energy production.

#### Interpreting the Extra Trees regressor with SHAP values

The Extra Trees Regressor combined with SHAP values offers a more flexible and insightful approach compared to traditional econometric approaches^39,40^. The Extra Trees Regressor can capture complex, nonlinear interactions between variables without explicit specification. SHAP values play a crucial role in interpreting the predictions made by ensemble models like the Extra Trees Regressor. They decompose the ensemble’s output by assigning each feature an importance value that quantifies its average marginal contribution across all possible combinations of features. This fair and consistent attribution allows us to understand precisely how each feature influences the model’s predictions, considering linear and nonlinear effects and interactions among variables. By quantifying the magnitude and direction of each feature’s effect, SHAP values indicate whether a feature increases or decreases the predicted value, enhancing the interpretability of complex models.

While traditional panel regression provides average effects with clear statistical significance tests, SHAP values with the Extra Trees Regressor offer detailed insights into how each feature influences predictions, including capturing nonlinear effects and interactions. Moreover, SHAP values make ensemble models interpretable by quantifying the impact of each feature on individual predictions^39^. This approach requires fewer assumptions about the data’s underlying structure and is more adaptable to complex and high-dimensional data, which is common in economic studies.

#### Utilizing SHAP dependence plots

We utilize SHAP dependence plots, supported by the python SHAP library^38^, to visualize the dependence of the model’s predictions on individual features. These plots show how variations in a feature’s value affect its SHAP values, illustrating the relationship between the feature and the model’s output. To explicitly demonstrate the impact of each variable, we fit logarithmic or exponential curves to the plots. In addition, we compare SHAP values across groups of countries using color-coded plots or separate subgroup analyses. This comparison reveals distinct patterns in how different country groups influence the model’s predictions, highlighting subgroup-specific effects and offering a more comprehensive understanding of the model’s behavior across varied contexts.

## Data Availability

All data are publicly available and were obtained from the following sources: data on CO$_2$ emissions https://ourworldindata.org/CO$_2$-and-greenhouse-gas-emissions; readiness and vulnerability are from the ND-GAIN database https://gain.nd.edu/our-work/country-index/; Chinn-Ito index (financial openness) https://web.pdx.edu/~ito/Chinn-Ito_website.htm; GDP https://data.worldbank.org/indicator/NY.GDP.MKTP.CD; Domestic credit to the private sector (as % of GDP) https://databank.worldbank.org/metadataglossary/world-development-indicators/series/FD.AST.PRVT.GD.ZS. We used an updated version of the climate-related financial policy database^26^, which originally included data up to 2020. Further data was collected until 2023, as explained in the Data section.

## Code availability

The code to replicate the study is available from the authors upon request.
