# Publisher Correction: Evaluating climate-related financial policies’ impact on decarbonization with machine learning methods

**DOI:** 10.1038/s41598-025-00837-2

**Published:** 2025-05-13

**Authors:** Paola D’Orazio, Anh-Duy Pham

**Affiliations:** 1 Chair of Economics, Faculty of Economics and Business Administration, Chemnitz University of Technology, Thüringer Weg 7, 09126 Chemnitz, Germany; 2 Joint Lab Artificial Intelligence and Data Science, Osnabrück University, 49074 Osnabrück, Germany

Correction to: *Scientific Reports* 10.1038/s41598-025-85127-7, published online 11 January 2025

The original version of this Article contained errors in Fig. 7 and Fig. 8, where panels **A** and **C** in both figures were duplications of panels **B** and **D**.

The original Fig. 7 and Fig. 8 and accompanying legends appear below.

**Fig. 7:**
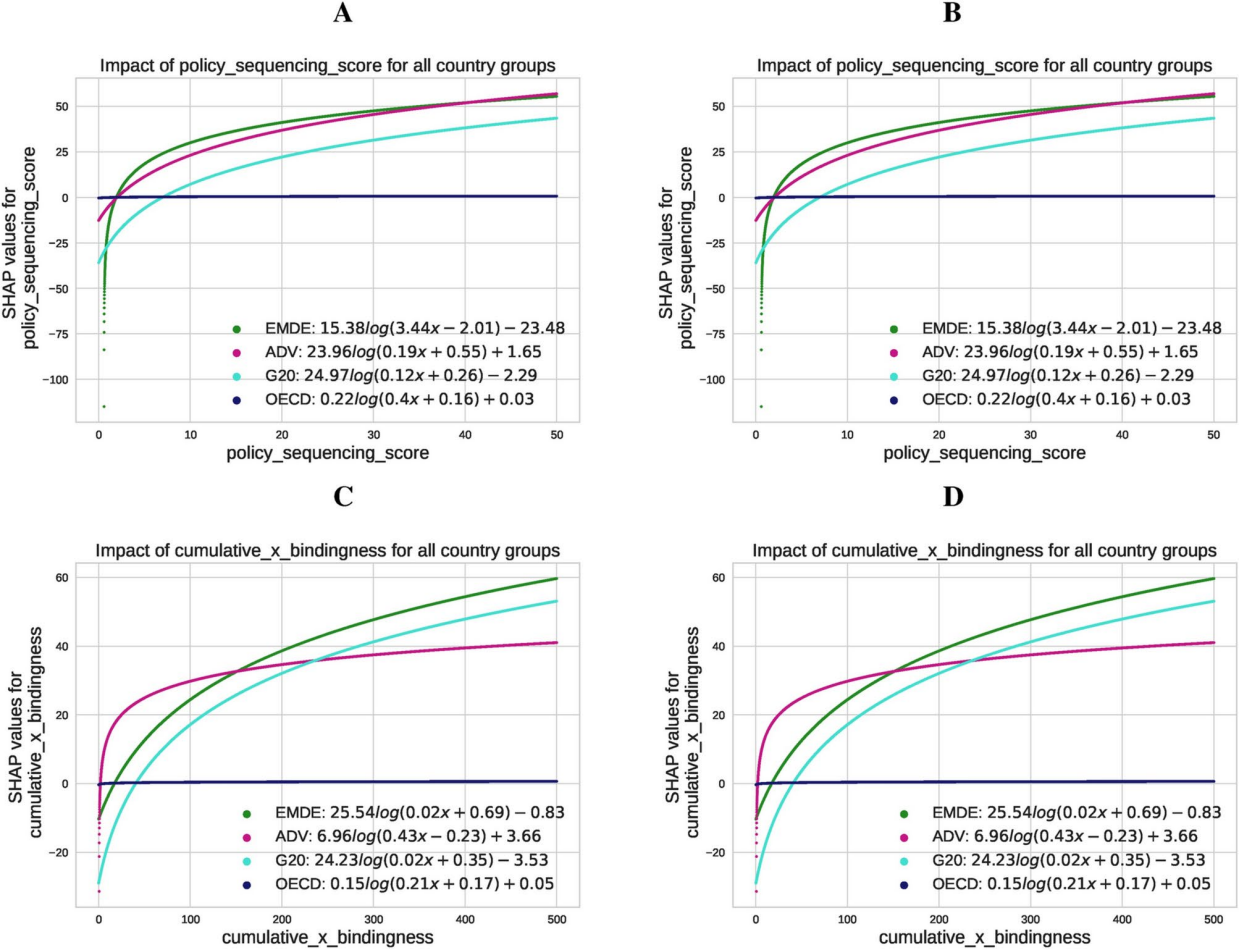
Fitted SHAP values: impact of the policy sequencing score and bindingness-weighted adoption on ${\text{CO}}_{2}$ emissions (Panels **A** and **C**) and renewable energy production (Panels **B** and **D**) across country groups (EMDE, ADV, G20, OECD). The results highlight differing trends based on structural, institutional, and economic contexts, with notable contrasts between EMDEs and advanced economies.

**Fig. 8:**
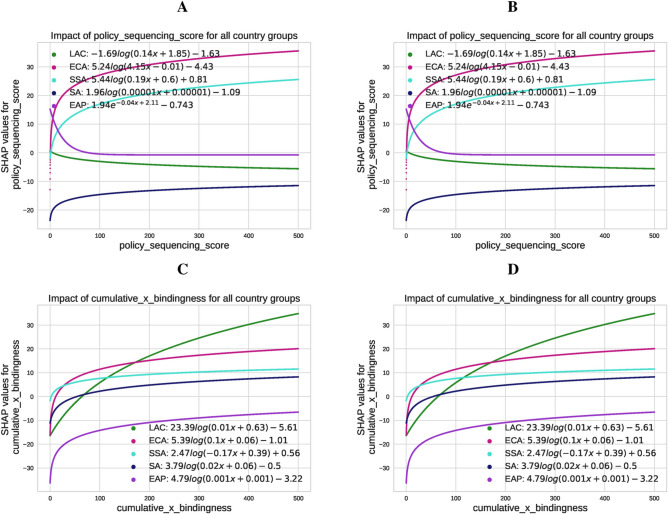
Fitted SHAP values illustrating the impact of the policy sequencing score and the bindingness-weighted adoption on ${\text{CO}}_{2}$ emissions and renewable energy production across different country groups. Panel (**A**) displays the SHAP values for the effect of the PSS score on ${\text{CO}}_{2}$ emissions for regions: Latin America and the Caribbean (LAC), Europe and Central Asia (ECA), Sub-Saharan Africa (SSA), South Asia (SA), and East Asia and the Pacific (EAP). Panel (**B**) shows the SHAP values for the PSS score’s impact on renewable energy production for the same regions. Panels C and D depict the SHAP values for the effect of cumulative bindingness-weighted policy adoption on ${\text{CO}}_{2}$ emissions (Panel **C**) and renewable energy production (Panel **D**) across these regions. The fitted SHAP values are calculated using logarithmic transformations, capturing region-specific variations in the influence of policy adoption and sequencing.

The original Article has been corrected.

